# Determinant Powers of Socioeconomic Factors and Their Interactive Impacts on Particulate Matter Pollution in North China

**DOI:** 10.3390/ijerph18126261

**Published:** 2021-06-09

**Authors:** Xiangxue Zhang, Yue Lin, Changxiu Cheng, Junming Li

**Affiliations:** 1 Key Laboratory of Environmental Change and Natural Disaster, Ministry of Education, Beijing Normal University, Beijing 100875, China; zxx@lreis.ac.cn; 2 State Key Laboratory of Earth Surface Processes and Resource Ecology, Beijing Normal University, Beijing 100875, China; 3 Department of Geography, The Ohio State University, Columbus, OH 43210, USA; lin.3326@buckeyemail.osu.edu; 4 National Tibetan Plateau Data Center, Beijing 100101, China; 5 School of Statistics, Shanxi University of Finance and Economics, Taiyuan 030006, China

**Keywords:** GeoDetector, long-term variations, PM_2.5_ concentrations, spatial autocorrelation, spatial heterogeneity

## Abstract

Severe air pollution has significantly impacted climate and human health worldwide. In this study, global and local Moran’s *I* was used to examine the spatial autocorrelation of PM_2.5_ pollution in North China from 2000–2017, using data obtained from Atmospheric Composition Analysis Group of Dalhousie University. The determinant powers and their interactive effects of socioeconomic factors on this pollutant are then quantified using a non-linear model, GeoDetector. Our experiments show that between 2000 and 2017, PM_2.5_ pollution globally increased and exhibited a significant positive global and local autocorrelation. The greatest factor affecting PM_2.5_ pollution was population density. Population density, road density, and urbanization showed a tendency to first increase and then decrease, while the number of industries and industrial output revealed a tendency to increase continuously. From a long-term perspective, the interactive effects of road density and industrial output, road density, and the number of industries were amongst the highest. These findings can be used to develop the effective policy to reduce PM_2.5_ pollution, such as, due to the significant spatial autocorrelation between regions, the government should pay attention to the importance of regional joint management of PM_2.5_ pollution.

## 1. Introduction

In recent years, problems related to air pollution have become increasingly severe worldwide and air pollution had a severe impact on climatic, economic development, and human health [1,2,3,4]. Of the atmospheric pollutants, particulate matter with an aerodynamic diameter of less than 2.5 μm (PM_2.5_) is the most harmful pollutant and has received increased attention recently [5,6,7]. PM_2.5_ can severely impact environmental conditions, reducing atmospheric visibility and including climate change [8]. Even more seriously, previous studies have demonstrated that severe PM_2.5_ pollution can cause pathological reactions in respiratory and cardiovascular systems and greatly increase mortality [9,10,11]. Due to rapid urbanization, these problems have become more prominent in China [12,13] and may not only lead to the migration of large populations but also to the increased consumption of energy [14]. Therefore, PM_2.5_ and related issues have received widespread research attention worldwide.

In early studies, more attention was paid to explore the driving factors of PM_2.5_ pollution, it has become increasingly apparent that socioeconomic and other pollutant factors would impact the accumulation and diffusion of PM_2.5_ worldwide [15,16]. Puentes et al. took Santiago, the capital of Chile, as an example, and used a bivariate regression prediction model to estimate the PM levels related to PM_2.5_ and PM_10_ at the same time. The results showed that the two pollutants have a strong relationship [17]. Due to the city being the spatial carrier of the above sources, one changes in response to the changes of others.

With the rapid urbanization, more research has involved socioeconomic factors such as population density [18], coal consumption [16], industrial structures [19], built-up areas [20], per capita gross domestic product (GDP) [21], and road density [16], as a means of determining which human activities contribute most to PM_2.5_ concentrations. For example, Cavieres et al. showed that there is severe air pollution in Santiago of Chile, which is mainly caused by anthropogenic activities that use combustion and may further exert adverse effects on human health [22]. Wu et al. constructed panel data models within an environmental Kuznets curve framework to investigate the correlations between per capita per capita gross domestic product (GDP) and PM_2.5_ concentrations; their result was an inverted U-shaped curve [21]. By employing a structural equation model, Jiang et al. demonstrated that PM_2.5_ concentrations in China were related to city size and the activities of both industries and residents [23]. Based on nonparametric additive regression models, Xu et al. found that, in China, there was an inverted-U shaped pattern between PM_2.5_ concentrations and economic activity, urbanization, number of private vehicles, and coal consumption [24].

Moreover, to detect and quantify the dominant driving factors of PM_2.5_ pollution, research studies have typically used linear models, such as spatial Durbin analyses [25], Bayesian hierarchical analyses [26], traditional econometrics models [27], panel data models [21], spatial lag/error models [28], geographic weighted regressions [29] and clustering methods [30]. For example, Du et al. indicated that PM_2.5_ concentrations were strongly correlated to GDP and built-up area [25]. Using a Pearson’s correlation analysis, Han et al. demonstrated that population size, built-up area, and the proportion of secondary industries had significant positive impacts on PM_2.5_ [20]_._ Moreover, Zhang et al. found a positive correlation between PM_2.5_ concentrations and population size using spatial interpolation [31]. These are just a few examples of the growing knowledge that socioeconomic factors have important impacts on PM_2.5_ pollution using a series of linear models.

However, it remains unclear which, among these potential factors, is dominant in terms of PM_2.5_ pollution in North China. Notably, the potential interactive impacts of these factors on PM_2.5_ pollution and whether or not either their determinant powers or interactions will remain the same over time are also currently unknown. Moreover, traditional methods are flawed in quantifying the interaction of factors that affect PM_2.5_. For example, the interaction of two factors can take many forms of coupling in reality, but in traditional regression methods, it is usually the product of two factors, and their ability to explain the heterogeneity of spatial stratification is poor. Therefore, in contrast to the various linear methods used in previous studies, non-linear models, such as GeoDetector, have been applied in this study to both quantify the determinant powers of socioeconomic factors and their interactive effects on PM_2.5_ concentrations in North China. That is, the aims of this study were to: (1) examine the evolution of hot/cold spots (i.e., regions becoming more/less polluted) in North China from 2000–2017, (2) identify the dominant factor(s) impacting PM_2.5_ concentrations each year, and (3) quantify the determinant powers and their interactive effects of socioeconomic factors on PM_2.5_ pollution over time using GeoDetector software for the measurement and attribution of stratified heterogeneity. The rest of this study is designed as follows: data sources and models applied in this study are introduced in Section 2, while the analysis results and key findings are mainly elaborated in Section 3. Section 4 then provides a discussion of the findings, and finally, Section 5 presents the study conclusions and policy recommendations.

## 2. Materials and Methods

### 2.1. Study Area

**Figure 1:**
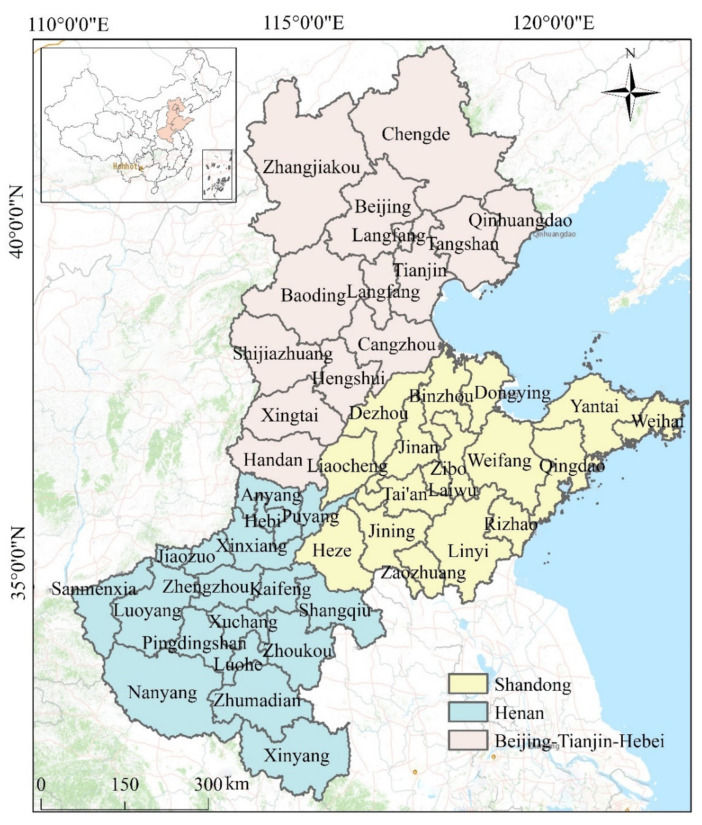
Spatial location of the study area.

North China comprises the provinces of Hebei, Henan, and Shandong, which consist of 48 cities, including Beijing and Tianjin (Figure 1). Compared with other regions in China, the urban population in this region suffers more severe PM_2.5_ pollution due to the presence of a large number of coal-based industries [32]. According to the Ministry of Environmental Protection of China, the air quality in 2015 of Baoding, Xingtai, Hengshui, Tangshan, Zhengzhou, Jinan, Handan, Shijiazhuang, Langfang, and Shenyang were the worst 10 cities, all these cities are located in the North China region, aside from Shenyang. Furthermore, the heating of most cities in this region places a great burden on the atmospheric environment, especially in winter. The area of North China is 543,000 km^2^; with a population of approximately 320 million, it is one of the most densely populated regions in China. In recent years, the accelerated growth of urbanization and industrialization in this region, means that energy consumption is enormous, and it has generated a large amount of atmospheric pollution, significantly impacting the atmospheric environment; this cannot be ignored. Therefore, the effective prevention and management of PM_2.5_ pollution in the area is crucial.

### 2.2. Data Sources

**Figure 2:**
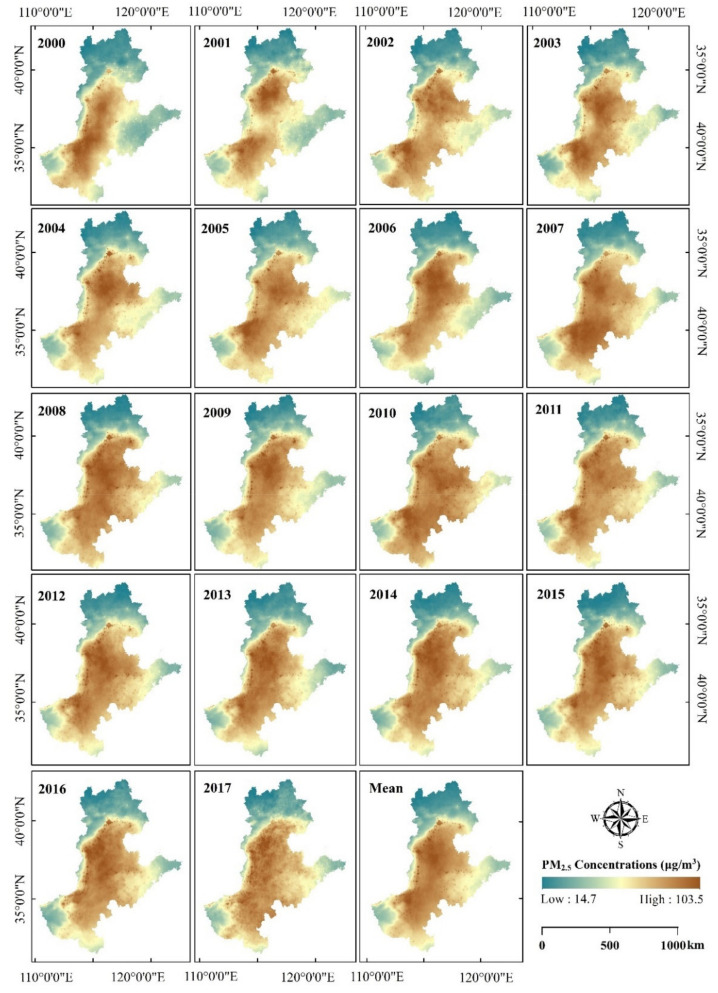
Spatiotemporal evolution of the annual mean PM_2.5_ concentrations from 2000–2017 in North China (data source: http://fizz.phys.dal.ca/~atmos/martin/ accessed on 20 May 2020). Based on the results of previous studies, the explanatory variables used in this study were: population density (PD), industrial output (IO), number of industries (NI), road density (RD), fossil fuel combustion (FC), and proportion of the non-agricultural population (UR) (Table 1). The UR is a traditional and commonly used proxy to represent urbanization. Additionally, the road data calculated in this study were obtained from Open Street Map [36], which maps urban roads and multi-segment highways. The line density tool in ArcGIS was then used to calculate RD in each city of the study area from 2000 to 2017.

The PM_2.5_ concentration data from 2000–2017 analyzed in this study were collected from the Atmospheric Composition Analysis Group of Dalhousie University [33], which has a spatial resolution of 0.1° × 0.1°. This dataset has great accuracy as it has been corrected with global station-based observation values based on the geographically weighted regression model, with an R^2^ value of 0.817 [34] and has been used in many studies [16,34,35]. Using the regional statistical tools in ArcGIS 10.3, the mean PM_2.5_ concentration data for each year in each city of the study area from 2000 to 2017 were then extracted (Figure 2).

### 2.3. Statistical Analyses

A global Moran’s *I* model was used to reveal whether or not the PM_2.5_ concentrations of a given region impacted its neighboring regions within the study period. Local indicators of spatial association (LISA) statistics were used to explore the hot/cold (high/low) spots of PM_2.5_ concentrations. The GeoDetector (www.geodetector.cn, accessed on 18 June 2020) model was then used to quantify the determinant powers and interactive effects of socioeconomic factors on PM_2.5_ concentrations.

First, in this study, a global Moran’s *I* model [37] was employed to test and quantify the spatial autocorrelation of PM_2.5_ concentrations from 2000–2017. The formula for this model is defined as follows:

(1) $$I=\frac{n}{\sum_{i=1}^{n}\sum_{j=1}^{n}w_{ij}}\frac{\sum_{i=1}^{n}\sum_{j=1}^{n}w_{ij}\left(y_{i}-\bar{y}\right)\left(y_{j}-\bar{y}\right)}{\sum_{i=1}^{n}{\left(y_{i}-\bar{y}\right)}^{2}},$$

where the *I* value ranges from −1 to 1 and the larger the absolute value of *I* is, the stronger the autocorrelation becomes; *n* is the number of cities; *w_ij_* is the spatial connection matrix, the value of which is 1 if polygons *i* and *j* are adjacent; and *y_i_*, *y_j_,* and $\bar{y}$ are the city-level mean values in cities *i*, *j*, and the study area, respectively. The statistic *I* was obtained in this study using 999 Monte Carlo permutations, and the significance of the *I* value was measured by the *p*-value (at the 5% significant level), which performed in GeoDa 1.16 software and was download from http://geodacenter.github.io/download.html (accessed on 18 June 2020), and the features of computer used is 64 bit.

Second, local indicators of spatial association (LISA) was used to analyze global spatial autocorrelation analysis, but it cannot present local differences in each city. Therefore, to detect the hot/cold spots of the study area, LISA statistics were used [38] according to the following formula:

(2) $$I_{i}=\frac{n\left(y_{i}-\bar{y}\right)\sum_{j=1}^{n}w_{ij}\left(y_{j}-\bar{y}\right)}{\sum_{i=1}^{n}{\left(y_{i}-\bar{y}\right)}^{2}},$$

where *y_i_*, *y_j_*, $\bar{y}$, and *w_ij_* are the same as in Equation (1). If *I_i_* > 0, a positive correlation existed between city *i* and the surrounding cities with similar levels of PM_2.5_ pollution. This facilitated the observation of spatial clusters (i.e., hot spots (high-high; HH) or cold spots (low-low; LL)). Conversely, a negative correlation was observed, it indicated the presence of spatial outliers (i.e., high-low, HL; low-high, LH).

All of these analyses were implemented in GeoDa 1.16 software and download from http://geodacenter.github.io/download.html (accessed on 18 June 2020), and the features of computer used is 64 bit.

Third, GeoDetector was used to quantify the determinant powers of socioeconomic factors and their interactive effects on PM_2.5_ concentrations. It was assumed that if a given factor (X) determined the response variable (Y), the response variable would present a similar spatial distribution to that of the factor [39,40,41]. It has been employed in the fields of geology, environment, health, disasters, and other many fields [42,43,44], as indicated in the following formula:

(3) $$q=1-\frac{\sum_{h=1}^{L}N_{h}\sigma_{h}^{2}}{N\sigma^{2}},$$

where the *q* value ranged from 0 to 1, indicating the determinant power of the factor. The larger the value of *q*, the stronger the determinant power of the factor. Here, *N* and *N_h_* represent the number of cities in the entire study area and stratum *h*, respectively; *σ^2^* and *σ_h_^2^* denote the variance of all cities in the study and stratum *h* and the study area was stratified into L strata, denoted by *h* = 1, 2,…, *L*.

**Figure 3:**
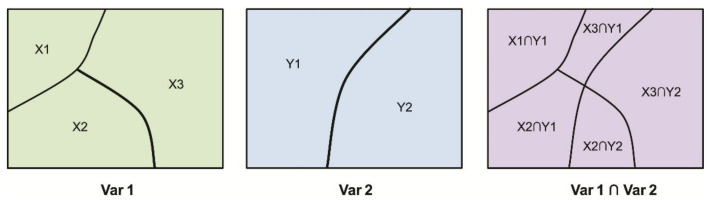
Principle of interactive effects.

The interactive effects of different factors (Xs) can also be quantified by the GeoDetector, which investigates whether or not the interactions of factors (X1∩X2) weakens or enhances the influence on Y by comparing the values of *q*(X1), *q*(X2), and *q*(X1∩X2) (Figure 3).

It is noteworthy that one of the advantages of GeoDetector is that it does not make linear assumptions for factors, such that the problem of multicollinearity is precluded. Therefore, adding or deleting variables will not impact the results. The GeoDetector tool was download from www.geodetector.cn (accessed on 10 July 2020) and implementation in Excel 2019 (Microsoft, Redmond, WA, USA) (the features of computer used is 64 bit).

## 3. Results

### 3.1. Spatiotemporal Analyses

The global Moran’s *I* values of PM_2.5_ concentrations from 2000–2017 were all positive and statistically significant at an *α*-level = 1% (*p* < 0.01) (Table 2). Notably, the values of *I* all exceeded 0.60, with a maximum value of 0.75 and a minimum of 0.61. This not only indicates that PM_2.5_ concentrations were significantly spatially autocorrelated in the study area (i.e., cities with high(low) PM_2.5_ concentrations tended to cluster near cities with high(low) values), but also that these autocorrelations remained high during the study period. Only the results at four time points are shown; however, they are representative of the full data (Figure S1). Most of the points shown in Figure S1 are found in the first and third quadrants, indicating that during the selected period, most cities exhibited significant and positive spatial autocorrelations.

**Figure 4:**
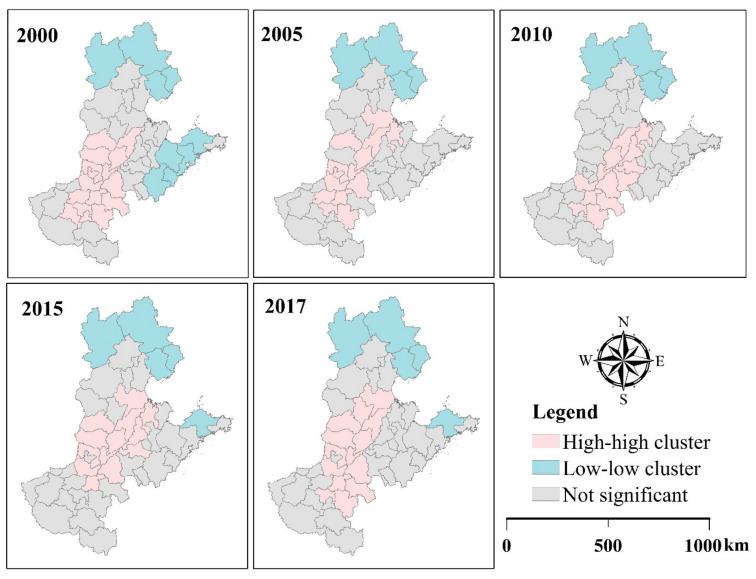
Local Moran’s *I* scatterplots for 2000, 2005, 2010, 2015, and 2017 (data source: http://fizz.phys.dal.ca/~atmos/martin/) (accessed on 15 March 2020).

The local Moran’s *I* value was used to identify the hot and cold spots of the PM_2.5_ concentrations in North China. Only the results at five time points, which are representative of the results, are shown here (Figure 4). There were no outliers (high-low or low-high) in the study area, meaning that no areas tended to aggregate with areas with different PM_2.5_ pollution concentrations.

Spatially, the risk of PM_2.5_ pollution was relatively high in southeastern Hebei, northern Henan, and western Shandong, while it was relatively low in northern Hebei and eastern Shandong. The frequency of clusters between 2000 and 2017 indicates that Xinxiang, Kaifeng, Dezhou, Xingtai, and Heze are mostly represented by HH clusters each year (≥16 out of 18), while Qinhuangdao and Chengde are represented by LL clusters each year (Figure S2). In general, stable and high concentrations of PM_2.5_ clusters were mainly found in southeastern Hebei, northern Henan, and western Shandong.

**Figure 5:**
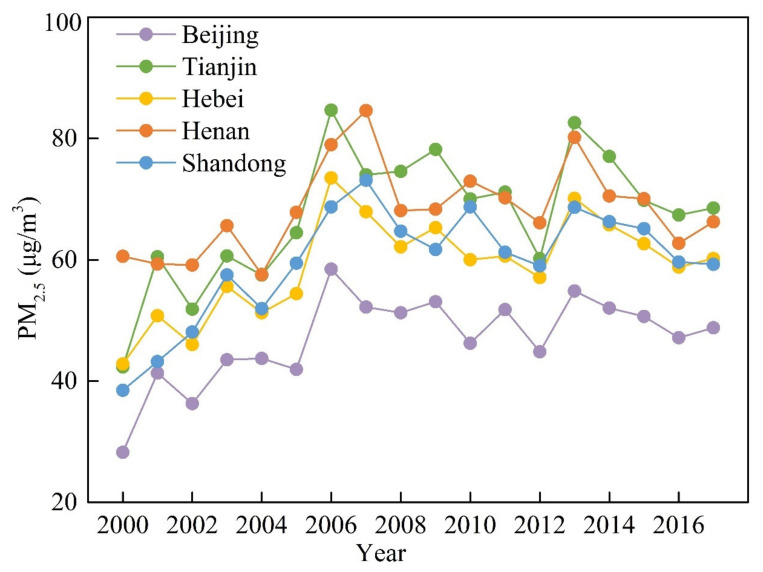
Temporal variations of PM_2.5_ concentrations from 2000–2017 (data source: http://fizz.phys.dal.ca/~atmos/martin/) (accessed on 15 March 2020).

Temporally, the mean annual value of PM_2.5_ for each province was extracted to capture its temporal evolution, which showed an upward trend (Figure 5). Both temporal variations and regional differences were observed. Regionally, relatively higher PM_2.5_ concentrations were recorded in Tianjin and Henan from 2000–2007. In Beijing, it has been always low. As for temporal variation, there are also significant regional differences. For example, the annual mean PM_2.5_ concentrations in 2000 were 28.24, 42.30, 42.80, 60.59, and 38.48 μg/m^3^ in Beijing, Tianjin, Hebei, Henan, and Shandong, respectively, in which the PM_2.5_ concentrations in Tianjin, Hebei, and Henan were more than 40 μg/m^3^. Before 2006, PM_2.5_ concentrations in Beijing, Tianjin, and Hebei all showed obviously increasing trends, with peak values of 58.47, 84.66, and 73.50 μg/m^3^, respectively. However, slightly declining trends were observed between 2007 and 2012. In Henan and Shandong, PM_2.5_ concentrations also showed primarily increasing trends before 2007, reaching maxima of 84.59 and 73.13 μg/m^3^, followed by a nearly continuous decline from 2007 to 2012. Interannual fluctuations have been observed since values peaked in 2013 in Beijing, Tianjin, Hebei, Henan, and Shandong.

### 3.2. Impact Factors

The determinant powers (*q* values) of the impact factors from 2000–2017 were quantified using GeoDetector (Table 3). Among the five selected factors, PD showed the greatest determinant power for PM_2.5_ concentrations (*q* = 0.67), followed by IO (*q* = 0.60), NI (*q* = 0.56), RD (*q* = 0.53), FC (*q* = 0.36), and UR (*q* = 0.32). The *q* values presented here are the results for 2017. Of the selected factors, PD exhibited a remarkably dominant effect on PM_2.5_ concentrations, as demonstrated by its *q* values, which ranged from 0.27 to 0.67 between 2000 and 2017 (Table 3), which was consistent with the results of other studies, for example, Wang et al. demonstrated that PD had a critical effect, reflected in the accumulation of PM_2.5_ by region [18]; similar, Ding et al. showed that PD had the remarkable impact on PM_2.5_ concentrations [45].

The *q* values of RD indicated that traffic conditions also had an increasing impact on PM_2.5_ pollution. The *q* values for RD (Table 3) ranged from 0.29 to 0.65, indicating that it accounted for between 29% and 65% of PM_2.5_ concentrations. The factors, IO and NI, generated *q* values from 0.42 to 0.60 and from 0.26 to 0.56, respectively, denoting an increasing and significant determinant power of industrial activities on PM_2.5_ pollution. It is generally believed that industrial growth, as represented by IO and NI, has a non-negligible impact on the degradation of the environment (Table 3). The *q* values of UR revealed that the influence of urbanization on PM_2.5_ pollution was increasing and non-negligible, ranging from 0.19 to 0.47, indicating that urbanization accounted for between 19 to 47% of annual mean PM_2.5_ concentrations. The *q* values of FC showed that the influence of fossil fuel combustion on PM_2.5_ pollution was increasing and non-negligible, ranging from 0.17 to 0.45, indicating that fossil fuel combustion accounted for between 17 to 45% of annual mean PM_2.5_ concentrations (Table 3).

**Figure 6:**
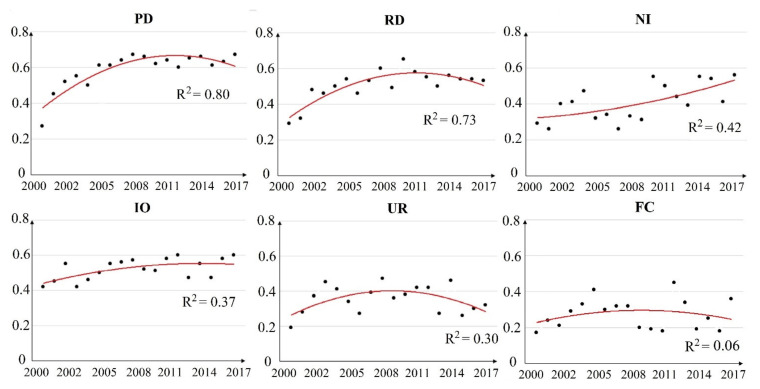
Scatter plots with trend lines of *q* values for each impact factor from 2000–2017.

Furthermore, the scatter plots for the selected periods display the *q* values with quadratic polynomials to fit the trend lines, as well as the labeled R^2^ values with which to explore the changing patterns of these driving factors (Figure 6). PD, RD, UR, and FC all showed a tendency to first increase and then decrease from 2000–2017. Both NI and IO showed increasing trends in the long-term, indicating that industrial activities have an increasing influence on PM_2.5_ pollution (Figure 6).

**Figure 7:**
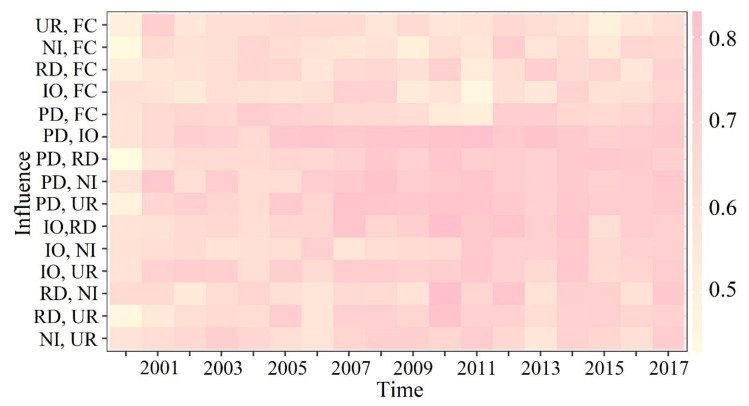
Heatmap of interactive effects from 2000–2017.

Moreover, in total, fifteen pairs of interactions among the five studied variables from 2000–2017 were calculated using the GeoDetector (Figure 7). It was found that the interactive effects of two random factors were greater than the effect of each individual factor, (i.e., the interactions between paired factors had a greater impact on PM_2.5_ pollution than any single factor). The strongest interaction between factors changed according to the year. For example, *q*(PD∩IO) showed the strongest interaction on five occasions between 2000 and 2017, ranging from 0.59–0.82, indicating that the interaction between PD and IO had the strongest impact on the spatial pattern of PM_2.5_ pollution. Over the study period, *q*(PD∩IO), *q*(PD∩NI), and *q*(RD∩NI) had the strongest interactions five, four, and four times, respectively, indicating that population, traffic, and industrial development had increasing interactive effects on the spatial pattern of PM_2.5_ pollution.

**Figure 8:**
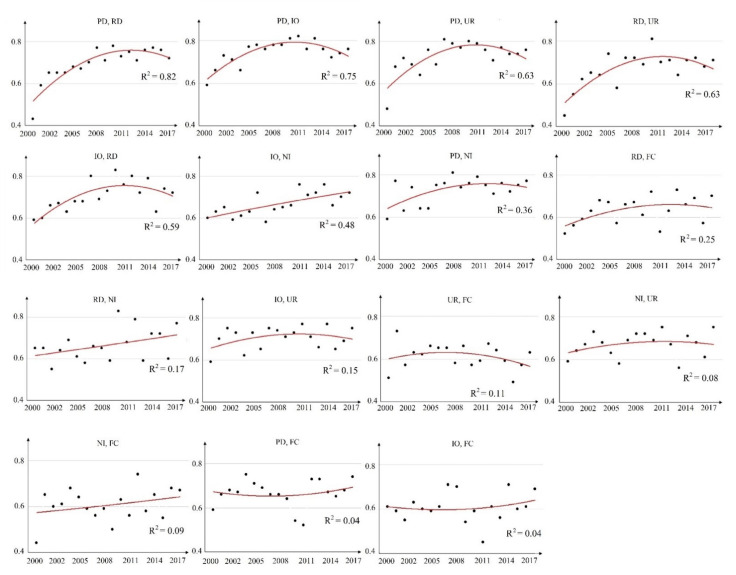
Scatter plots with trend lines of *q* values for each pair of interactive effects from 2000–2017.

Between 2000 and 2017, the scatter plots in Figure 8 show the interactive effects with quadratic polynomials used to fit trend lines and R^2^ to explore the changing patterns of interactive effects for the driving factors. The interactive effects of (PD, RD), (PD, IO), (PD, UR), (RD, UR), (IO, RD), (PD, NI), (IO, UR), (RD, FC), (UR, FC), and (NI, UR) essentially showed a tendency to first increase and then decrease (Figure 8). Both (IO, NI), (IO, FC), (PD, FC), (NI, FC), and (RD, NI) showed long-term increasing trends, meaning that the influence of these factors on PM_2.5_ concentrations has gradually increased.

## 4. Discussion

In recent years, problems related to air pollution, especially PM_2.5_, have become increasingly prominent and have attracted widespread attention. North China, as one of the most densely populated and rapidly urbanizing regions in China, with millions of residents and rapid economic development, has suffered severely from PM_2.5_ pollution. In this study, a global Moran’s *I* model was used to examine the spatial autocorrelation of PM_2.5_ concentrations from 2000–2017. A LISA model was utilized to explore the hot and cold spots of PM_2.5_ concentrations and determinant powers of socioeconomic factors and their interactive impacts on PM_2.5_ pollution was quantified using GeoDetector. The results indicate that the cities with high pollution risks are mainly distributed in Xinxiang, Kaifeng, Dezhou, Xingtai, and Heze and exhibit significant spatial autocorrelation. Furthermore, the determinant power of PD remained high during the study period, and the interaction between PD and IO was the strongest on five occasions between 2000 and 2017.

In this study, the temporal evolution of PM_2.5_ concentrations in North China from 2000–2017 was examined and found to have an overall increasing trend. A potential reason for this may be that China has experienced rapid urbanization in recent years, especially in the study area, which is the most concentrated region in terms of industry (e.g., steel, and vehicle use) [32]. It is notable that these industries consume tremendous amounts of fossil fuels and inevitably generate large amounts of industrial emissions. Moreover, in the process of urbanization, populations are increasingly concentrated in urban regions, and the demands of transportation and consumption of energy continue to expand with increasing population densities.

Significant and positive spatial autocorrelations existed in the PM_2.5_ concentrations analyzed here. Hot spots were mainly concentrated in southeastern Hebei, northern Henan, and western Shandong, and specifically in cities such as Xinxiang, Kaifeng, Dezhou, Xingtai, and Heze, to which more attention should be paid. Cold spots were distributed across northern Hebei, southern Henan, and eastern Shandong, in cities such as Qinhuangdao, Chengde, and Yantai.

Many prior studies have demonstrated that socioeconomic factors significantly impact the spatiotemporal patterns of PM_2.5_ pollution, and it is widely accepted that PD is a remarkable factor influencing PM_2.5_ concentrations. Similarly, in this study, the determinant power of PD was the strongest (e.g., *q* = 0.67 in 2017), which is widely consistent with the results of previous studies. For example, Ding et al. showed that PD had the strongest impact on PM_2.5_ concentrations [45], while Lou et al. demonstrated that PD had a critical effect, reflected in the accumulation of PM_2.5_ by region [46]. Wang et al. also found that PD exerts a remarkable effect on PM_2.5_ concentrations [18]. Hinojosa et al. indicated that population was correlated with PM_2.5_ concentration using data from Mexico City [47]. The results of the current study demonstrate that anthropogenic emissions are the key factors leading to significant increases in PM_2.5_ concentrations in North China between 2000 and 2017. Anthropogenic emissions are also a fundamental source of secondary aerosols of PM_2.5_ [48]. Dense populations also consume greater amounts of non-renewable energy, which is considered to be the main source of PM_2.5_ [28].

In this study, IO was found to have a significant impact on the spatial distribution of PM_2.5_ concentrations, as has been shown in previous studies. For example, Yang et al. demonstrated that IO had a significant influence on the spatial pattern of PM_2.5_ concentrations in China [16], and Wang et al. determined that industrial development would increase industrial emissions, resulting in inevitable increases in PM_2.5_ concentrations [18]. In another study, Zhang et al. suggested that the proportion of secondary industry is the decisive factor in terms of PM_2.5_ pollution, as industrial activities are the main emission sources, including energy-intensive and high-polluting mining, construction, and manufacturing, which emit exhaust gases and dust into the atmosphere that can form PM_2.5_ pollution through direct or secondary reactions [19]. Additionally, some regions lack proper management and fail to implement effective energy-saving measures and environmental protection policies. In such regions, PM_2.5_ emissions have greatly increased. Industry still affects economic and social development in China.

Furthermore, a strong association between RD and PM_2.5_ concentrations was found, according to the indicator of traffic conditions (*q* = 0.53), which is consistent with previous research. Yang et al. demonstrated that in China, traffic conditions (depicted by RD) have a significant impact on the spatial patterns of PM_2.5_ concentrations [16]. Similarly, Wu et al. determined that traffic conditions, represented by the proportion of road area, exerted a remarkable effect on PM_2.5_ pollution [21], while Zhang et al. showed that the increasing length of highways would significantly raise the health risks related to PM_2.5_ in eastern China [19]. Oliveira et al. demonstrated that emissions of light-duty vehicles have impacted on the PM_2.5_ pollution using data from Brazil [49]. This may occur because rapid urbanization is generally accompanied by a huge demand for buildings, road networks, and other public infrastructure, which directly increase energy consumption and the amount of dust (e.g., emission of vehicles, road and building dust) [21]. Highly accessible roads also indicate increased vehicle usage [50]. Therefore, RD has a remarkable impact on the accumulation and diffusion of PM_2.5_.

In assessing the interactions of all tested factors, they all presented non-linear enhanced effects, that is, after interaction, the effect is greater than that of the single factor. Moreover, GeoDetector revealed significantly increasing interactive effects, of which, PD and IO were the strongest on five occasions between 2000 and 2017. Because socioeconomic factors are closely related to anthropogenic and industrial emissions, changes over the past few decades to the original ecological environment and human living conditions have increased anthropogenic and industrial emissions that will further promote PM_2.5_ pollution under the influence of rapidly increasing global urbanization. For instance, socioeconomic activities mainly interact from a spatial perspective, in the ecological environment, and with human settlements and farmland systems, which also strengthen PM_2.5_ pollution [51]. Therefore, the interaction of socioeconomic factors will amplify the impact of PM_2.5_ to an extent.

## 5. Conclusions, Limitations, and Future Research

In this study, a global Moran’s *I* was used to examine the spatial autocorrelation of PM_2.5_ pollution in North China from 2000–2017. Local indicators of spatial association statistics were then used to identify hot and cold spots before the determinant powers of socioeconomic factors and their interactive effects were finally quantified using GeoDetector. The PM_2.5_ concentrations in the study area exhibited an increasing trend from 2000 to 2017. Additionally, the concentrations showed significant and stronger global spatial autocorrelation. Hot spots were mainly concentrated in the cities of Xinxiang, Kaifeng, Dezhou, Xingtai, and Heze, while cold spots were mainly distributed in the cities of Qinhuangdao and Chengde. PD was shown to have the most decisive influence on the spatial pattern of PM_2.5_ pollution, and the interactive effect of PD and IO was the strongest for many times during the period. Between 2000 and 2017, the impacts of PD, RD, UR, and FC trended to first increase and then decrease, while those of NI and IO showed an obviously increasing trend. Finally, the interactive effects of most factors appeared to first increase and then decrease, while the others showed a significant increasing trend. In total, these findings provide a clearer picture of the socioeconomic factors responsible for PM_2.5_ pollution across cities in North China, which has significant meaning and may be used to guide policy development and remediation measures in the future and mean that more specific control strategies need to be developed for different regions to successfully control and reduce PM_2.5_ pollution.

Some limitations of this study require mentioning. First, we only considered socioeconomic impact factors in this study, while environmental conditions, such as air temperature and atmospheric humidity, and other pollutant factors, such as PM_10_, were omitted, in the future research, more attention should be paid to analyze the association between other pollutants and PM_2.5_ pollution. Second, the association between compositional data and impact factors can be considered in the future research [52]. Third, the data used in our analyses were binned by city (spatially) and by year (temporally), likely introducing some uncertainties. In addition, the accuracy of raw PM_2.5_ data is considerably high, but it also exists uncertainty in different regions. Thus, data with high spatiotemporal resolution used in further studies might provide high accuracy and efficiency of the quantification of socioeconomic impacts on PM_2.5_ pollution.

## Figures and Tables

**Table 1:** Descriptions of the indicators for socioeconomic factors.

Variables	Definitions	Units	Data Sources
Population	Population density (PD)	10^4^ person/km^2^	China City Statistical Yearbook (http://data.cnki.net/NewHome/index) (accessed on 16 May 2020)
Industry	Industrial output (IO)	10^6^ Yuan/km^2^	
	Number of industries (NI)	/	
Traffic	Road density (RD)	Km/km^2^	Open Street Map (https://www.openstreetmap.org) (accessed on 20 May 2020)
Urbanization	Proportion of non-agricultural population (UR)	%	China City Statistical Yearbook (http://data.cnki.net/NewHome/index) (accessed on 16 May 2020)
Energy use	Fossil fuel combustion (FC)	10^4^ m^3^	China City Statistical Yearbook (http://data.cnki.net/NewHome/index) (accessed on 16 May 2020)

**Table 2:** Global Moran’s *I* of PM_2.5_ concentrations from 2000–2017.

Year	Moran’s *I*	Year	Moran’s *I*
2000	0.75	2009	0.61
2001	0.66	2010	0.64
2002	0.67	2011	0.65
2003	0.68	2012	0.71
2004	0.62	2013	0.70
2005	0.67	2014	0.63
2006	0.66	2015	0.67
2007	0.72	2016	0.67
2008	0.61	2017	0.67

**Table 3:** The *q* values of each influence factor from 2000 to 2017.

Time	PD	IO	NI	RD	UR	FC
2000	0.27	0.42	0.29	0.29	0.19	0.17
2001	0.45	0.45	0.26	0.32	0.28	0.24
2002	0.52	0.55	0.40	0.48	0.37	0.21
2003	0.55	0.42	0.41	0.46	0.45	0.29
2004	0.50	0.46	0.47	0.50	0.41	0.33
2005	0.61	0.50	0.32	0.54	0.34	0.41
2006	0.61	0.55	0.34	0.46	0.27	0.30
2007	0.64	0.56	0.26	0.53	0.39	0.32
2008	0.67	0.57	0.33	0.60	0.47	0.32
2009	0.66	0.52	0.31	0.49	0.36	0.20
2010	0.62	0.51	0.55	0.65	0.38	0.19
2011	0.64	0.58	0.50	0.58	0.42	0.18
2012	0.60	0.60	0.44	0.55	0.42	0.45
2013	0.65	0.47	0.39	0.50	0.27	0.34
2014	0.66	0.55	0.55	0.56	0.46	0.19
2015	0.61	0.47	0.54	0.54	0.26	0.25
2016	0.63	0.58	0.41	0.54	0.30	0.18
2017	0.67	0.60	0.56	0.53	0.32	0.36

## Data Availability

The data presented in this study are available on request from the corresponding author.

## Supplementary Materials

The following are available online at https://www.mdpi.com/article/10.3390/ijerph18126261/s1, Figure S1: Global Moran’s *I* values of PM_2.5_ concentrations in 2000, 2005, 2010, and 2017, Figure S2: Frequency of high-high (HH) and low-low (LL) clusters from 2000–2017.
