# Supplementary material for: Determinant Powers of Socioeconomic Factors and Their Interactive Impacts on Particulate Matter Pollution in North China
Source: Int J Environ Res Public Health. 2021 Jun 9;18(12):6261. doi: 10.3390/ijerph18126261 (PMC8296047; doi:10.3390/ijerph18126261)
Supplement: Supplementary file 1 [file ijerph-18-06261-s001.zip › ijerph-1235875-Supplementary.pdf]

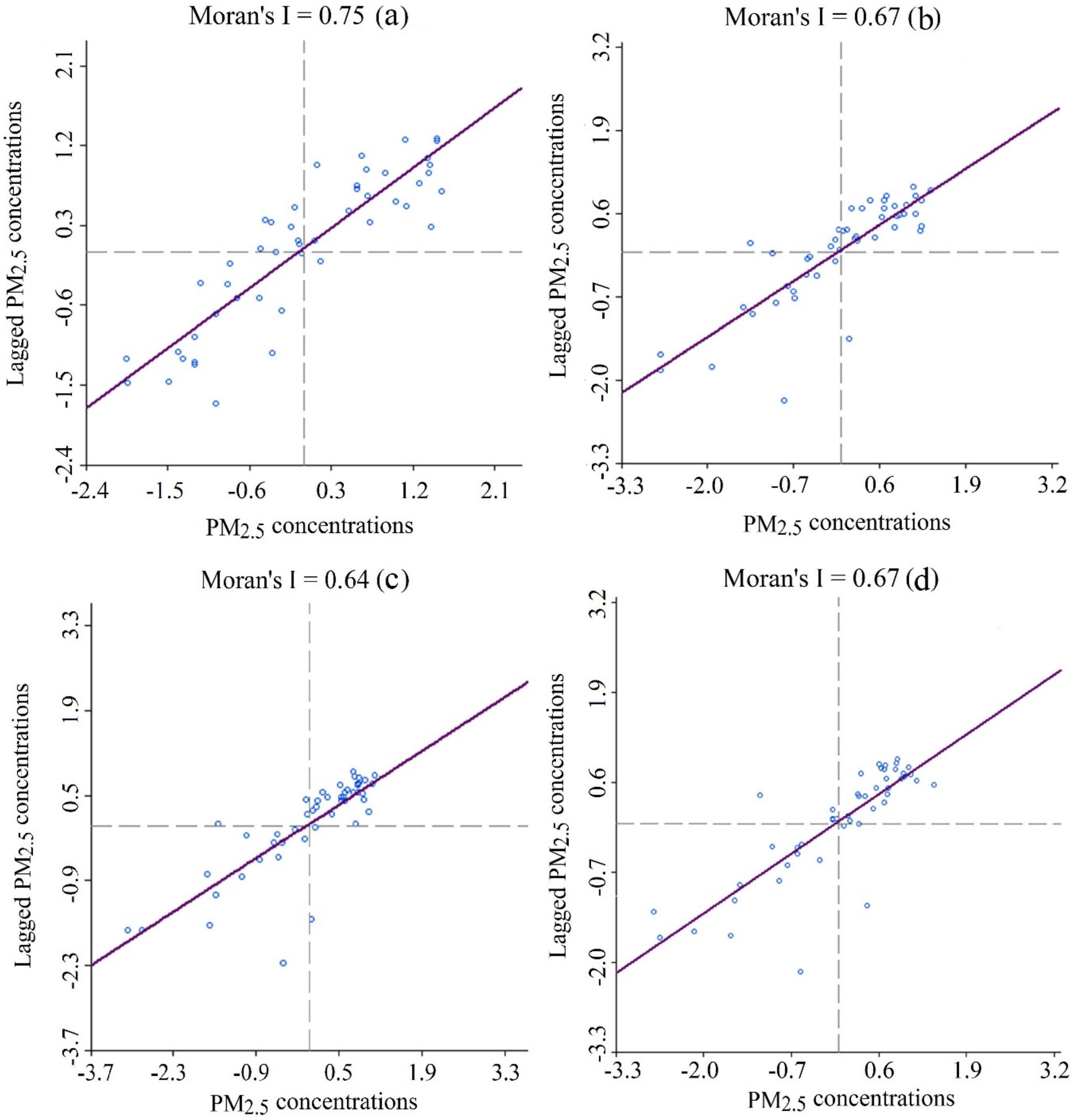

Figure S1. Global Moran's *I* values of $PM_{2.5}$ concentrations in 2000, 2005, 2010, and 2017. Note: Labels (**a**), (**b**), (**c**), and (**d**) correspond with the years 2000, 2005, 2010, and 2017.

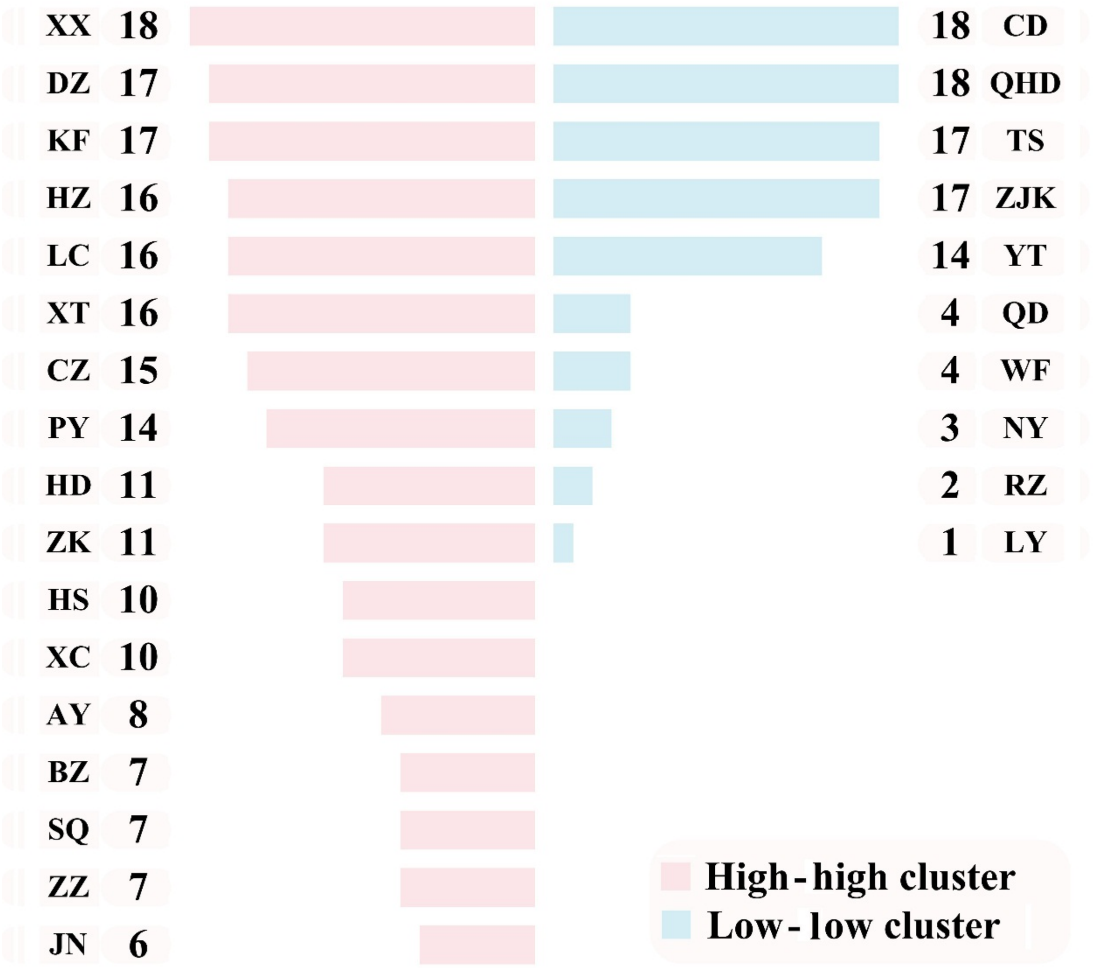

Figure S2. Frequency of high-high (HH) and low-low (LL) clusters from 2000-2017. Abbreviation: XX, Xinxiang; DZ, Dezhou; KF, Kaifeng; HZ, Heze; LC, Liaocheng; XT, Xingtai; CZ, Cangzhou; PY, Puyang; HD, Handan; ZK, Zhoukou; HS, Hengshui; XC, Xuchang; AY, Aayang; BZ, Binzhou; SQ, Shangqiu; ZZ, Zhengzhou; JN, Jinan; CD, Chengde; QHD, Qinhuangdao; TS, Tangshan; ZJK, Zhangjiakou; YT, Yantai; QD, Qingdao; WF, Weifang; NY, Nanyang; RZ, Rizhao; LY, Linyi.

Note: HH with frequency less than 5 times are not displayed in the Figure.
